# Dietary risk analysis of heavy metal and metalloid pollution in rice: case study of Nanning City in China

**DOI:** 10.3389/fpubh.2026.1781196

**Published:** 2026-04-10

**Authors:** Peng Li, Siyan Li, Jing Zhang, Liting Cen, Qi Li, Hui Li

**Affiliations:** 1 Department of Public Health, Guangxi University of Science and Technology, Liuzhou, Guangxi, China; 2 Department of Public Health, Youjiang Medical University for Nationalities, Baise, Guangxi, China; 3 Nanning Center for Disease Control and Prevention, Nanning, Guangxi, China; 4 School of Civil and Architectural Engineering, Guangxi University of Science and Technology, Liuzhou, Guangxi, China

**Keywords:** cadmium, dietary risk, heavy metal, Monte Carlo probabilistic assessment, rice

## Abstract

**Background:**

Heavy metal contamination of rice represents an important dietary exposure pathway in southern China. This study evaluated heavy metal and metalloid contamination and associated health risks in rice from Nanning City.

**Methods:**

A total of 1,844 rice samples collected between 2014 and 2020 were used for probabilistic risk assessment, and 538 recent samples (2019–2020) were analyzed to characterize contamination levels. Deterministic and Monte Carlo approaches were applied to assess non-carcinogenic and carcinogenic risks.

**Results:**

Among four elements analyzed (Cd, Pb, Hg, and inorganic As), cadmium was the predominant contaminant, with a maximum concentration of 1.28 mg/kg (6.4 times the national limit) and an exceedance rate of 16.36% in recent samples. The combined hazard index (HI) was 1.13, indicating potential non-carcinogenic concern. The total carcinogenic risk (CCR) was 1.75 × 10^−5^. Monte Carlo simulation showed a mean THQ of 0.61, with 15.8% of the simulated population exceeding THQ = 1. Sensitivity analysis identified cadmium concentration as the primary determinant of exposure risk.

**Conclusion:**

Cadmium remains the dominant contributor to dietary risk from rice in Nanning City. Targeted monitoring and risk management in high-exposure areas are warranted.

## 1 Introduction

Rice is a staple food in the traditional Chinese diet, and China is the world's largest producer of rice, with cultivation spanning nearly every province. According to (1), rice production in China reached 6,916.9 kilograms per hectare in 2017. The safety of rice is of critical, as this crop can accumulate heavy metals and metalloids, including cadmium, lead, arsenic, chromium, and mercury, during its growth cycle. Among these contaminants, cadmium exhibits the highest enrichment capacity. Chronic consumption of rice contaminated with heavy metals and metalloids may adversely affect multiple organ systems, including the liver, kidneys, bones, lungs, and the immune system.

Previous studies have highlighted the health risks associated with dietary heavy metal and metalloids exposure. Previous studies have reported (2) that lead exposure contributes significantly to the onset of chronic kidney disease of uncertain etiology. This finding described itai-itai disease as a severe manifestation of chronic cadmium poisoning (3).

The present study measured mercury content in rice from 15 provinces and found that methylmercury is readily absorbed by mammals (4); although current mercury levels in rice across China were found to pose relatively low risks to the general population, long-term methylmercury exposure remains a concern due to its potential effects on the central nervous system.

Globally, concern regarding dietary heavy metal and metalloid exposure is increasing. For instance, the presence of heavy metals and metalloids in rice flour consumed by hypertensive populations in Nigeria (5). In Southeast Asian countries, where rice and rice products are dietary staples, heavy metals and metalloids contamination can have long-term health implications (6). In China, cadmium contamination is predominantly concentrated in the southern regions (7). A study analyzing 1,528 rice samples from Nanning collected between 2014 and 2018 revealed widespread heavy metal and metalloid contamination, with particularly high cadmium levels (8).

Historically, dietary assessments of heavy metals and metalloids often relied on point estimates, which did not account for variations in consumption or environmental pollution. Modern risk assessments now combine both carcinogenic and non-carcinogenic analyses, comparing estimated daily intake (EDI) with reference doses such as provisional tolerable weekly intake (PTWI) established by the Joint FAO/WHO Expert Committee on Food Additives (JECFA). Advanced statistical and probabilistic methods, including Monte Carlo simulations, have been increasingly applied in heavy metal and metalloids risk assessments (9). In China, Lu Ping employed the K-medoids algorithm to cluster data for dietary analysis of heavy metals in rice (10). Florence Mhungu et al. conducted a cumulative analysis of heavy metals and metalloids in the Guangzhou diet using relative potency molecular integration and exposure margin modeling (11). Some researchers have also suggested the development of new probabilistic models for heavy metal and metalloids risk assessments (9, 12).

In Guangxi, early industrialization and factory proliferation contributed to persistent heavy metal pollution. Despite China's promotion of green production and the principle that “green water and green mountains are as valuable as mountains of gold and silver,” heavy metal contamination remains a concern, particularly in the context of residents' diets. Assessing dietary risks from heavy metals and metalloids in Nanning City can provide a scientific foundation for mitigating contamination-related health hazards.

This study employed Geographic Information System (GIS) technology to visualize the spatial distribution of heavy metals and metalloids in rice across urban areas of Nanning. For elements detected at the highest concentrations, both point estimation and Monte Carlo probabilistic assessments were conducted to evaluate the dietary exposure risk to local residents. This approach transforms deterministic risk assessments into stochastic models, enhancing the scientific rigor and reliability of the results.

## 2 Methods

### 2.1 Study area and rice sample collection

This study was conducted in Nanning City, Guangxi Province, China, a major rice-producing region in southern China characterized by intensive rice cultivation and high dietary reliance on rice. Rice sampling was carried out continuously from 2014 to 2020 as part of a regional food safety surveillance program. A total of 1,844 locally produced rice samples were collected over the study period. Rice samples were obtained from three primary sources, including local households, agricultural markets, and supermarkets, to reflect typical rice consumption patterns of local residents. In addition, 180 paddy rice samples were collected from local households for comparative analysis.

To ensure clarity of study design, different subsets of samples were used for specific analytical purposes. Specifically, 538 samples collected during 2019–2020 (including 358 rice samples and 180 paddy samples) were used for detailed heavy metal and metalloid determination and exceedance assessment, reflecting the most recent contamination status. In contrast, all 1,844 locally produced rice samples collected between 2014 and 2020 were incorporated into the Monte Carlo probabilistic risk assessment to capture long-term variability in cadmium exposure.

Sampling locations covered 15 administrative districts and counties of Nanning City, which were categorized into three types: six suburban counties (Binyang, Long'an, Shanglin, Mashan, Hengxian, and Wuming), six urban districts (Xixiangtang, Jiangnan, Liangqing, Qingxiu, Xingning, and Yongning), and three development zones (ASEAN Economic and Technological Development Zone, High-tech Industrial Development Zone, and Jiangnan Economic and Technological Development Zone).

Within each district or county, five townships (east, south, west, north, and central locations) were selected using a plum-blossom sampling strategy to ensure spatial representativeness. Approximately 20% of natural villages within each township were then randomly selected, and rice samples were collected with approximately even distribution across villages. This hierarchical sampling strategy was designed to achieve comprehensive spatial coverage of rice-producing areas throughout the study region.

### 2.2 Dietary survey of local residents

A dietary survey was conducted among 524 residents from 300 rice-farming households in the study area (232 males and 292 females; mean age 55.14 ± 8.67 years). Individual rice consumption was assessed using a 24-h dietary recall combined with a food frequency questionnaire (FFQ).

Rice intake was recorded as raw rice equivalent, consistent with the format used in national food contamination risk monitoring and dietary exposure assessments in China. This approach ensured direct compatibility between dietary intake data and heavy metal concentrations measured in raw rice samples. Individual body weight information was collected during the survey and used for exposure assessment. The mean daily rice consumption was 227.8 ± 18.24 g/person/day, with a median intake of 231.56 g/person/day, which is higher than the national average (176.6 g/day, 2010–2012 survey), comparable to rural areas (212.9 g/day), and lower than the provincial average in Guangxi (361 g/day).

### 2.3 Health risk assessment

#### 2.3.1 Deterministic health risk assessment

##### 2.3.1.1 Average daily dose (ADD)

Dietary exposure to heavy metals through rice consumption was quantified using the average daily dose (ADD), calculated as:


$$\begin{array}{l} ADD=\frac{C\;\times \;IR}{BW} \end{array}$$ (1)

Where *C* represents the Cd concentration in rice (mg/kg), IR is the daily rice ingestion rate (kg/day), and BW is the body weight (kg).

##### 2.3.1.2 Non-carcinogenic risk assessment

Non-carcinogenic health risks were evaluated using the target hazard quotient (THQ) and hazard index (HI):


$$\begin{array}{lll} THQ & = & \frac{ADD}{RfD} \end{array}$$ (2)

$$\begin{array}{lll} HI & = & \sum THQ \end{array}$$ (3)

Where RfD represents the oral reference dose (mg/kg·day) for each element. A THQ < 1 or HI ≤ 1 indicates negligible non-carcinogenic risk, whereas THQ ≥1 or HI >1 suggests a potential health concern.

##### 2.3.1.3 Carcinogenic risk assessment

For elements classified as carcinogenic, individual carcinogenic risk (CR) and cumulative carcinogenic risk (CCR) were calculated as:


$$\begin{array}{lll} CR & = & ADD\times SF \end{array}$$ (4)

$$\begin{array}{lll} CCR & = & \sum CRi \end{array}$$ (5)

Where SF is the cancer slope factor (kg·day/mg). CR or CCR values < 1.0 × 10^−6^ indicate low carcinogenic risk; values between 1.0 × 10^−6^ and 1.0 × 10^−4^ indicate a potential carcinogenic risk that warrants attention; values >1.0 × 10^−4^ indicate a high carcinogenic risk.

##### 2.3.1.4 Estimated monthly intake (EMI) of cadmium

Long-term exposure to cadmium was further evaluated using the estimated monthly intake (EMI):


$$\begin{array}{l} EMI=\frac{ADD\times ED\times EF}{AT} \end{array}$$ (6)

Where ED is the exposure duration (years), EF is the exposure frequency (365 days/year), and AT is the averaging time (days). EMI values were compared with the Provisional Tolerable Monthly Intake (PTMI) for cadmium (25 μg/kg BW) recommended by JECFA to evaluate long-term health risks.

#### 2.3.2 Probabilistic health risk assessment (Monte Carlo simulation)

To account for variability and uncertainty in cadmium exposure, probabilistic health risk assessment was conducted using Monte Carlo simulation (5,000 iterations) with Latin Hypercube Sampling. Cadmium concentrations in rice (*n* = 1,844, collected from 2014–2020) were fitted to multiple probability distributions, and the lognormal distribution was selected based on goodness-of-fit tests, including the Akaike Information Criterion (AIC). Rice consumption data were modeled using a triangular distribution derived from dietary survey results. A total of 5,000 iterations were performed to estimate the population-level distributions of cadmium intake and THQ values, as well as the probability of THQ ≥1. Within the Monte Carlo framework, sensitivity analysis was conducted to quantify the relative contribution of input variables to variability in THQ estimates.

The reference doses (RfD), cancer slope factors (SF), and corresponding exposure metrics used in the health risk assessment are summarized in Table 1.

**Table 1:** Reference doses (RfD) and slope factors (SF) for heavy metals and metalloids and exposure metrics.

Chemical	Type	RfD [mg/(kg·d)]	SF (kg·d/mg)	Exposure metrics
Arsenic (As)	Carcinogenic	0.0003	1.5	THQ, HI, CR, CCR
Cadmium (Cd)	Carcinogenic	0.0005	6.1	THQ, HI, EMI
Lead (Pb)	Carcinogenic	0.0014	0.0085	THQ, HI, CR, CCR
Mercury (Hg)	Non-carcinogenic	0.0003	——	THQ, HI

RfD, reference dose; SF, slope factor; “—” indicates non-carcinogenic. Exposure Metrics: THQ, Target Hazard Quotient; HI, Hazard Index; CR, Carcinogenic Risk; CCR, Cumulative Carcinogenic Risk; EM, Estimated Monthly Intake.

### 2.4 Determination of heavy metals in rice

Rice and paddy samples were air-dried, dehusked when necessary, ground into powder, and passed through a 100-mesh sieve to ensure sample homogeneity. The processed samples were stored in polyethylene containers at 4 °C in the dark prior to analysis.

For the determination of total arsenic (As), cadmium (Cd), and lead (Pb), 0.5 g of homogenized sample was accurately weighed into a microwave digestion vessel, followed by the addition of 8 mL of concentrated nitric acid (HNO_3_). Samples were pre-digested at 100 °C for 1 h and subsequently digested using a microwave digestion system (MARS6, CEM, USA). After digestion, the solution was heated at 140 °C to remove excess acid and diluted to 10 ml with ultrapure water. Elemental concentrations were then determined using an inductively coupled plasma mass spectrometer (ICP-MS, iCAP Q, Thermo Fisher Scientific, USA). Mercury (Hg) was measured using a direct mercury analyzer (DMA-80, Milestone, Italy), in which 0.1 g of homogenized sample was directly introduced into the instrument for analysis.

Inorganic arsenic was determined using high-performance liquid chromatography coupled with atomic fluorescence spectrometry (HPLC-AFS, SA-20, Beijing Jitian Instrument Co., China) equipped with a Hamilton anion-exchange column (250 mm × 4 mm) and a guard column (10 mm × 4 mm). For extraction, 1.0 g of sample was mixed with 10 mL of 0.15 mol/L nitric acid and heated in a 90°C water bath for 2.5 h with shaking every 30 min. After cooling to room temperature, the extract was centrifuged at 8,000 rpm for 15 min, and the supernatant was filtered through a 0.45 μm membrane filter prior to chromatographic analysis.

Quality assurance and quality control procedures included reagent blanks, duplicate analyses (10% of samples), certified reference materials, and spike-recovery tests. Calibration curves were established using multi-element standard solutions, and the correlation coefficients (R^2^) of the calibration curves were all greater than 0.999. The limits of detection (LOD) were 0.0040 mg/kg for As, 0.0030 mg/kg for Cd, 0.0040 mg/kg for Pb, and 0.0050 mg/kg for Hg. The limits of quantification (LOQ) were defined as 10 times the LOD values. A certified reference material of rice flour [GBW(E)100359] was analyzed simultaneously to verify analytical accuracy, and the measured values were consistent with the certified values. For atomic fluorescence detection of arsenic species, the detection wavelength was set at 193.7 nm.

### 2.5 Statistical analysis

Data processing was performed using Excel 2010, and descriptive statistics were calculated using SPSS 24.0. Chi-square tests examined differences among groups, with significance at *P* < 0.05. Spatial distribution maps of Cd levels in rice were generated using ArcGIS 10.5.

### 2.6 Reference standards

Testing followed the National Risk Monitoring Workbook for Food Contamination and Harmful Factors. Samples were evaluated according to the National Food Safety Standard—Maximum Levels of Contaminants in Foods (13). A sample was considered unqualified if one or more contaminants exceeded the established limits. Detection limits were as follows: Pb < 0.050 mg/kg, Cd < 0.0030 mg/kg, total Hg < 0.010 mg/kg. Exceedance criteria for rice were defined as: Pb > 0.2 mg/kg, Cd > 0.2 mg/kg, inorganic As > 0.2 mg/kg, and total Hg > 0.02 mg/kg. The standard was updated in 2022 (14); however, the maximum permissible limits for cadmium, lead, mercury, and inorganic arsenic in rice remained unchanged. Therefore, application of GB 2762-2017 in this study is methodologically appropriate.

## 3 Results and analysis

In a total of 538 rice and paddy samples collected during 2019–2020, the detection rates of lead (Pb), cadmium (Cd), inorganic arsenic (iAs), and total mercury (Hg) were 53.90%, 99.07%, 24.91%, and 99.81%, respectively. These samples were used to characterize the contamination status and exceedance levels of heavy metals and metalloids in locally produced rice.

Among the four elements analyzed, cadmium exhibited the highest exceedance rate (16.36%), indicating that Cd is the dominant contaminant of concern in the study area. The maximum Cd concentration reached 1.28 mg/kg, which is 6.4 times higher than the national permissible limit (14) (Tables 2, 3). Notably, exceedances were not limited to marginal cases; a proportion of samples exhibited substantially elevated concentrations, suggesting the presence of high-contamination hotspots in certain areas. For concentrations below the limit of detection (LOD), values were substituted with one-half the LOD, following standard practice in dietary exposure assessment. Overall, these results indicate that cadmium exceedance is not only frequent but also variable in magnitude, with certain samples far exceeding the regulatory threshold, thereby potentially contributing disproportionately to dietary exposure.

**Table 2:** Overall detection of heavy metals in rice and paddy grown in Nanning, 2019–2020.

Testing project	*n* (samples)	Positive detections (*n*)	Detection rate (%)	Maximum concentration (mg/kg)	Mean ±SD (mg/kg)	LOD (mg/kg)	Exceedances (*n*)	Exceedance rate (%)	Regulatory limit (mg/kg)
**Lead (Pb)**	538	290	53.90	1.77	0.073 ± 0.187	< 0.0040	11	2.04	>0.2
**Cadmium (Cr)**	538	533	99.07	1.28	0.124 ± 0.137	< 0.0030	88	16.36	>0.2
**Inorganic As**	538	134	24.91	0.5	0.168 ± 0.087	< 0.0500	34	6.32	>0.2
**Mercury (Hg)**	538	537	99.81	0.038	0.008 ± 0.006	< 0.0005	7	1.30	>0.02

**Table 3:** Comparison of heavy metal exceedances in rice and paddy grown in Nanning, 2019–2020.

Testing program	Paddy (*n* = 180)		Rice (*n* = 358)		*χ^2^*	*P*
	Exceedances (*n*, %)	Mean ±SD (mg/kg)	Exceedances (*n*, %)	Mean ±SD (mg/kg)		
**Lead (Pb)**	3 (1.67%)	0.053 ± 0.066	8 (2.23%)	0.0389 ± 0.225	0.19	>0.05
**Cadmium (Cr)**	24 (13.33%)	0.109 ± 0.118	64 (17.88%)	0.0120 ± 0.143	1.81	>0.05
**Inorganic As**	32 (17.8%)	0.122 ± 0.007	2 (0.56%)	0.0029 ± 0.023	59.99	< 0.01
**Mercury (Hg)**	4 (2.22%)	0.008 ± 0.006	3 (0.84%)	0.0073 ± 0.006	1.79	>0.05
**Total**	60 (33.3%)	–	75 (21.0%)	–	9.77	< 0.01

### 3.1 Distribution of the number of townships with excessive cadmium in rice

**Figure 1:**
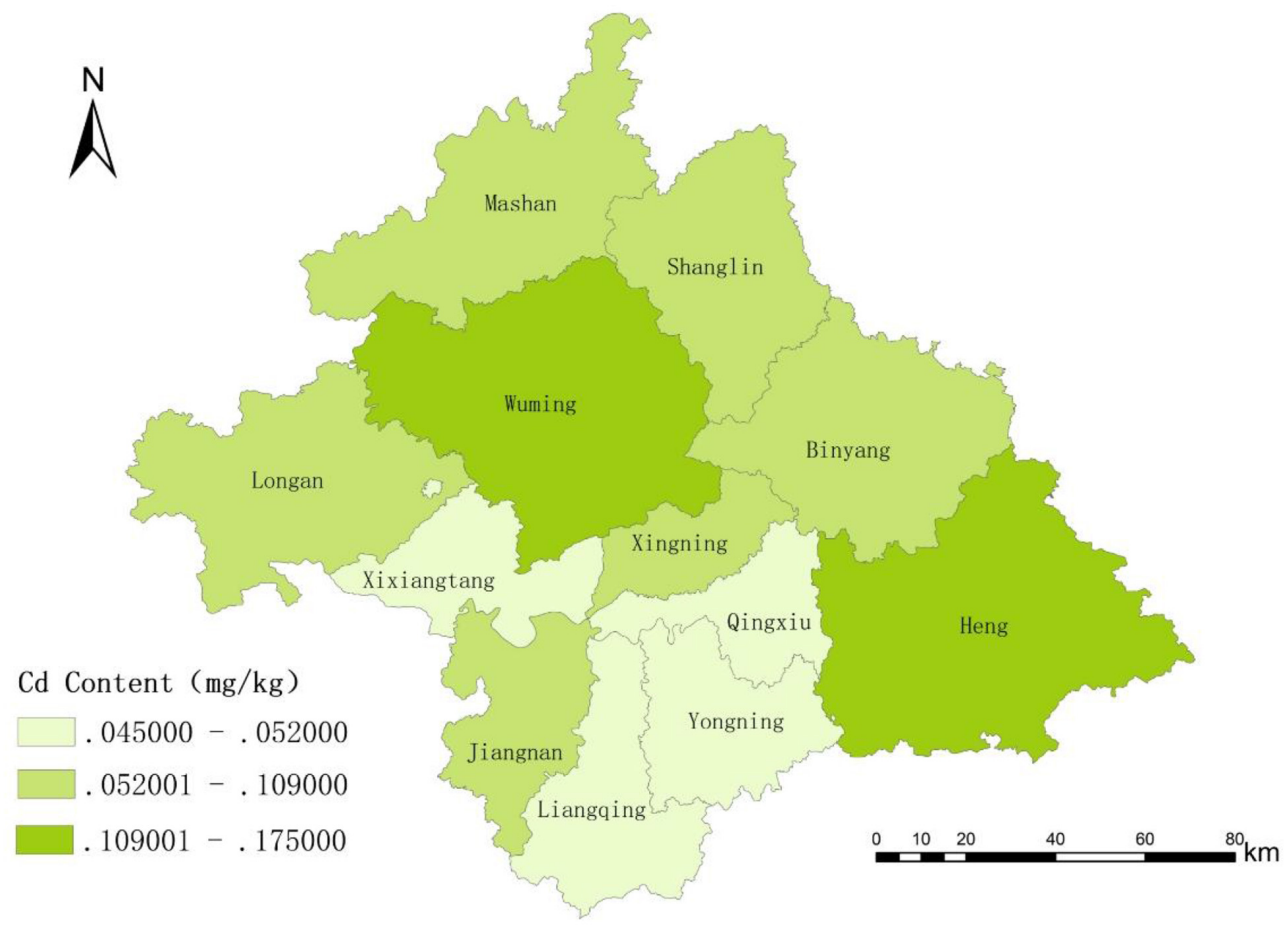
Distribution of townships with Cd detection value in rice in Nanning city.

At the township level, Long'an County, Mashan County, and Shanglin County showed relatively higher proportions of townships with cadmium exceedances in rice. The Cadmium concentration (P75) in rice from Mashan, Wuming, Shanglin, and Binyang counties was greater than the limit value (Table 4, Figure 1).

**Table 4:** Number of townships with cadmium exceedance in cultivated rice across counties/districts of Nanning, 2019–2020.

County (district)	Townships tested (*n*)	Exceeding townships (*n*)	P25 (mg/kg)	P50 (mg/kg)	P75 (mg/kg)
Long'an County	10	7	0.03	0.08	0.17
Hengxian County	11	7	0.03	0.07	0.21
Shanglin County	13	7	0.06	0.15	0.38
Mashan County	17	6	0.04	0.10	0.18
Wuming County	11	6	0.05	0.11	0.22
Binyang County	17	6	0.07	0.12	0.20
Liangqing district	9	6	0.02	0.06	0.13
Jiangnan district	9	5	0.03	0.09	0.19
XiXiangtang area	13	2	0.02	0.07	0.13
Yongning district	7	1	0.05	0.19	0.10
Xingning district	5	1	0.02	0.05	0.10
Qingxiu district	7	1	0.05	0.12	0.18
Total	129	55	0.05	0.09	0.18

### 3.2 Dietary survey of rice farmers

A total of 300 households in rice-growing areas with 524 individuals were surveyed. Among the participants, 232 were male and 292 were female, with an average age of 55.14 ± 8.67 years. The average intake of cooked rice and rice product was 227.8 ± 18.24 g/person-day, with a median of 231.56 g/person-day. This intake was higher than the national average for urban and rural residents (~176.6 g/standard person-day from 2010 to 2012~ 176.6 g/person/day, 2010–2012). The intake was also between the levels reported for general rural areas (212.9 g/person-day) and poor rural areas (240.5 g/person-day) and lower than the Guangxi provincial average of 361 g/person-day (2010–2012 survey).

### 3.3 Health risk assessment of heavy metals and metalloids in locally grown rice

#### 3.3.1 Non-carcinogenic risks (cadmium, lead, mercury, inorganic arsenic)

Based on the reported intake of cooked rice and rice products (median: 231.56 g/person·day):

Cadmium: ADD = 0.000449 mg/kg-d; THQ = 0.898 (< 1), indicating no significant non-carcinogenic risk.

Lead: ADD = 0.000146 mg/kg-d; THQ = 0.104 (< 1), negligible non-carcinogenic risk.

Total mercury: ADD = 0.0000273 mg/kg-d; THQ = 0.091 (< 1), negligible non-carcinogenic risk.

Inorganic arsenic: ADD = 0.0000108 mg/kg-d; THQ = 0.036 (< 1), negligible non-carcinogenic risk.

However, the combined Total Hazard Index (HI) for all four element was 1.13 (>1), indicating a potential non-carcinogenic health concern according to USEPA guidance, rather than confirmed adverse health effects. This finding suggests that further attention may be warranted, particularly for potentially sensitive subpopulations such as children and adolescents.

#### 3.3.2 Carcinogenic risk (lead, inorganic arsenic)

Based on rice and rice product intake:

Lead: ADD = 0.000227 mg/kg-d; CR = 1.24 × 10^−6^, indicating a low but non-negligible carcinogenic risk.

Inorganic arsenic: ADD = 0.0000108 mg/kg-d; CR = 1.63 × 10^−5^, indicating a moderate carcinogenic risk.

CCR = 1.75 × 10^−5^, meaning an estimated 17.5 cases per 1 million people could develop cancer, indicating a potential carcinogenic health concern. These results remain consistent under (14), as permissible limits have not substantially changed.

#### 3.3.3 Point assessment (cadmium)

**Figure 2:**
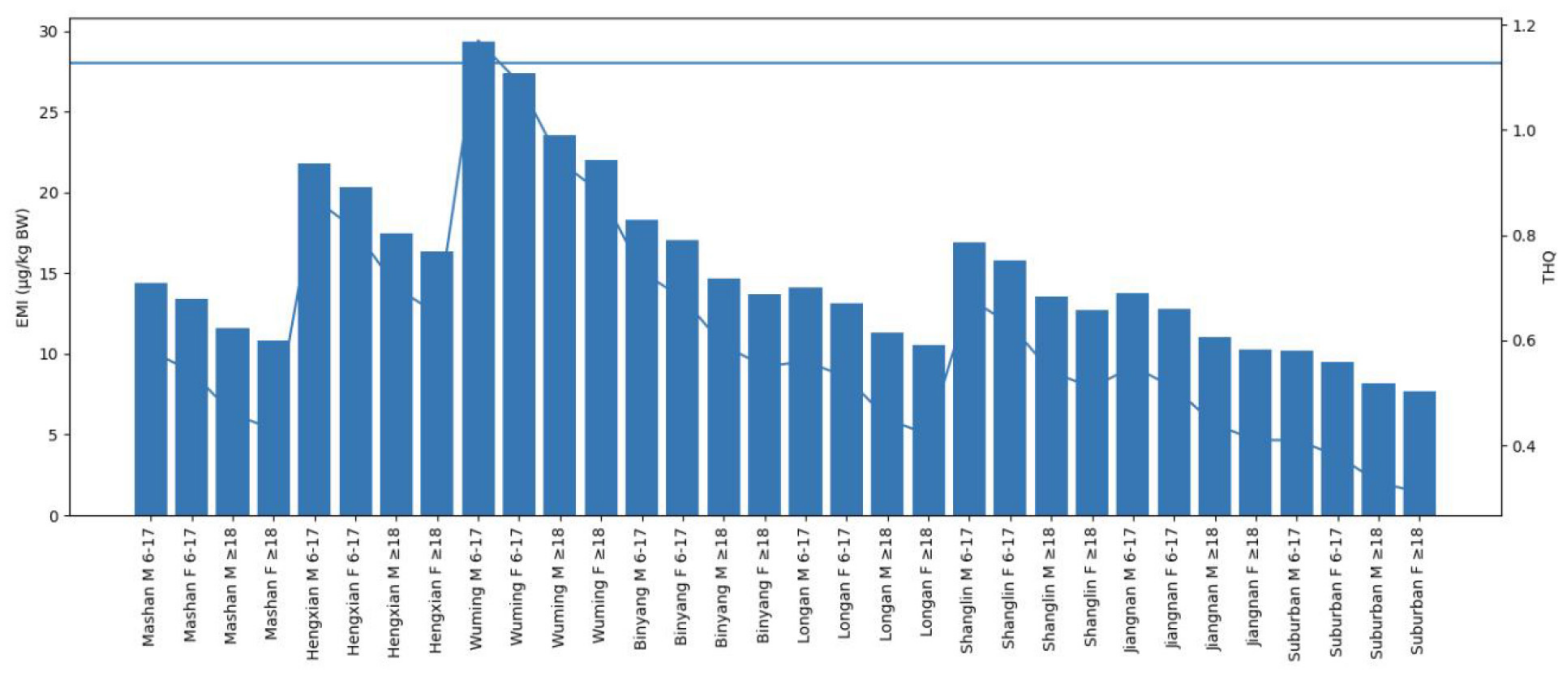
Estimated monthly intake (EMI) and hazard quotient (THQ) of cadmium exposure at the P50 level across different regions, age groups, and sexes in Nanning.

THQ values were generally below 1 across all regions and populations; however, in Wuming County, both males and females aged 6–17 years exhibited THQ values exceeding 1, indicating relatively elevated potential non-carcinogenic risk (Table 5 and Figure 2).

**Table 5:** Health risks of cadmium exposure at the P50 level in different counties/districts of Nanning, China.

District	Age group	Sex	EMI (μg/kg BW)	THQ50	District	Age group	Sex	EMI (μg/kg BW)	THQ50
**Mashan County**	6~17years	male	14.43	0.58	**Long'an County**	6~17years	male	14.09	0.56
		female	13.45	0.54			female	13.13	0.53
	≧18years	male	11.57	0.46		≧18years	male	11.31	0.45
		female	10.81	0.43			female	10.56	0.42
**Hengxian County**	6~17years	male	21.81	0.87	**Shanglin County**	6~17years	male	16.94	0.68
		female	20.32	0.81			female	15.79	0.63
	≧18years	male	17.5	0.7		≧18years	male	13.59	0.54
		female	16.34	0.65			female	12.69	0.51
**Wuming County**	6~17years	male	29.35	1.17	**Jiangnan district**	6~17years	male	13.75	0.55
		female	27.36	1.09			female	12.82	0.51
	≧18years	male	23.55	0.94		≧18years	male	11.04	0.44
		female	21.99	0.88			female	10.3	0.41
**Binyang County**	6~17years	male	18.28	0.73	**Nanning Suburban**	6~17years	male	10.23	0.41
		female	17.04	0.68			female	9.54	0.38
	≧18years	male	14.67	0.59		≧18years	male	8.21	0.33
		female	13.7	0.55			female	7.67	0.31

**Figure 3:**
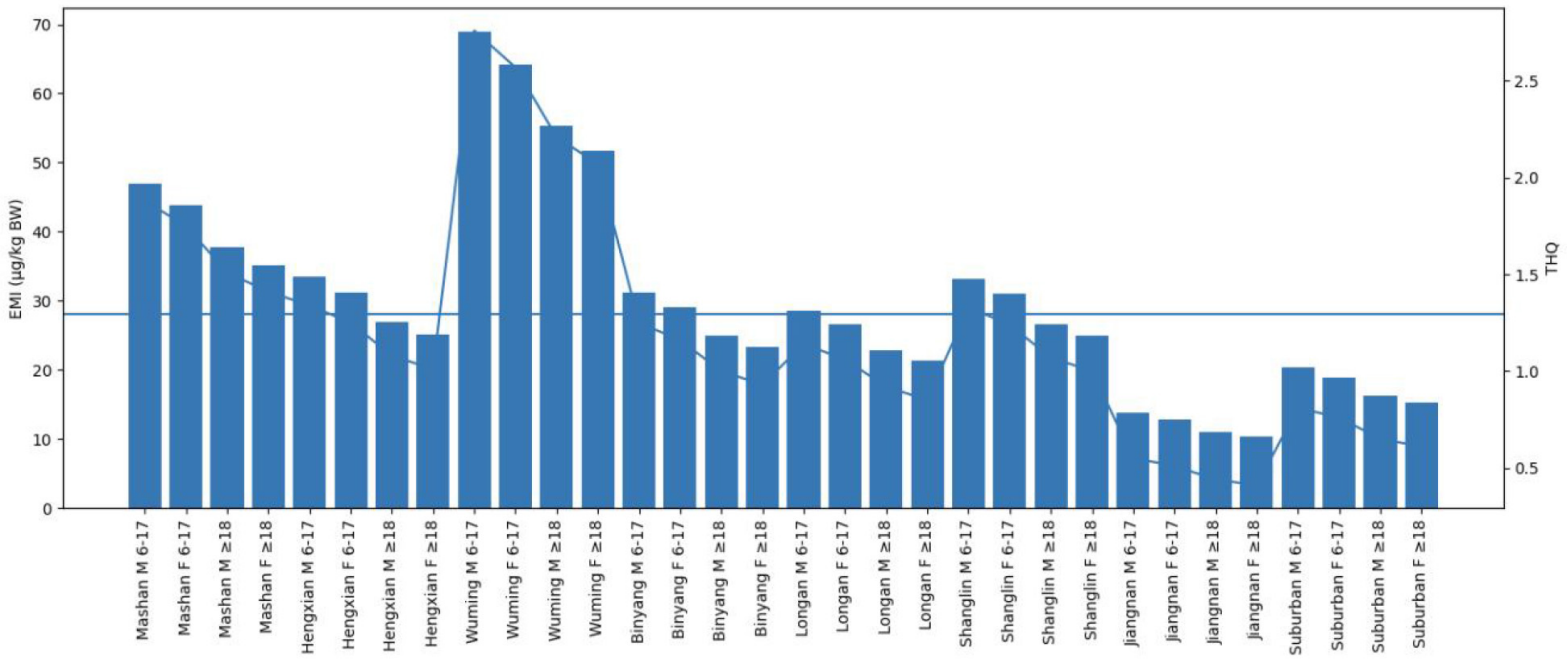
Estimated monthly intake (EMI) and hazard quotient (THQ) of cadmium exposure at the P75 level across different regions, age groups, and sexes in Nanning.

The estimated monthly exposure (EMI) for populations with high-dose exposures (P75) ranged from 1.85 to 64.25 μg/kg BW. THQ values exceeding 1 were observed in multiple counties, particularly in Wuming, Mashan, Hengxian, and Shanglin, suggesting spatial heterogeneity in non-carcinogenic risk (Table 6 and Figure 3).

**Table 6:** Health risks of cadmium exposure at the P75 level in different counties/districts of Nanning, China.

District	Age group	Sex	EMI (μg/kg BW)	THQ75	District	Age group	Sex	EMI (μg/kg BW)	THQ75
**Mashan County**	6~17years	male	46.97	1.88	**Long'an County**	6~17years	male	28.52	1.14
		female	43.77	1.75			female	26.58	1.06
	≧18years	male	37.68	1.51		≧18years	male	22.88	0.92
		female	35.18	1.41			female	21.36	0.85
**Hengxian County**	6~17years	male	33.55	1.34	**Shanglin County**	6~17years	male	33.21	1.33
		female	31.27	1.25			female	30.95	1.24
	≧18years	male	26.92	1.08		≧18years	male	26.65	1.07
		female	25.13	1.01			female	24.88	1
**Wuming County**	6~17years	male	68.94	2.76	**Jiangnan district**	6~17years	male	13.75	0.55
		female	64.25	2.57			female	12.82	0.51
	≧18years	male	55.31	2.21		≧18years	male	11.04	0.44
		female	51.65	2.07			female	10.3	0.41
**Binyang County**	6~17years	male	31.2	1.25	**Nanning Suburban**	6~17years	male	20.3	0.81
		female	29.08	1.16			female	18.92	0.76
	≧18years	male	25.03	1		≧18years	male	16.28	0.65
		female	23.37	0.93			female	15.2	0.61

#### 3.3.4 Monte Carlo probabilistic assessment method (Cd)

**Figure 4:**
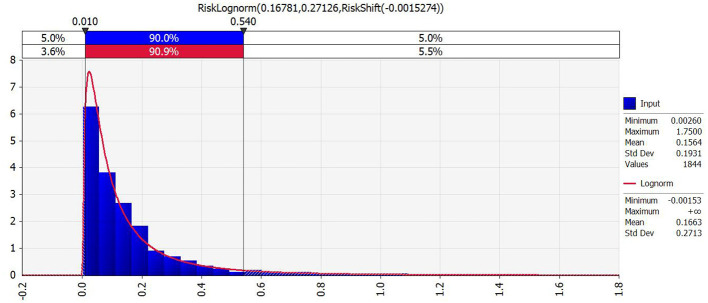
Fit Comparison for the distribution of cadmium in rice.

**Figure 5:**
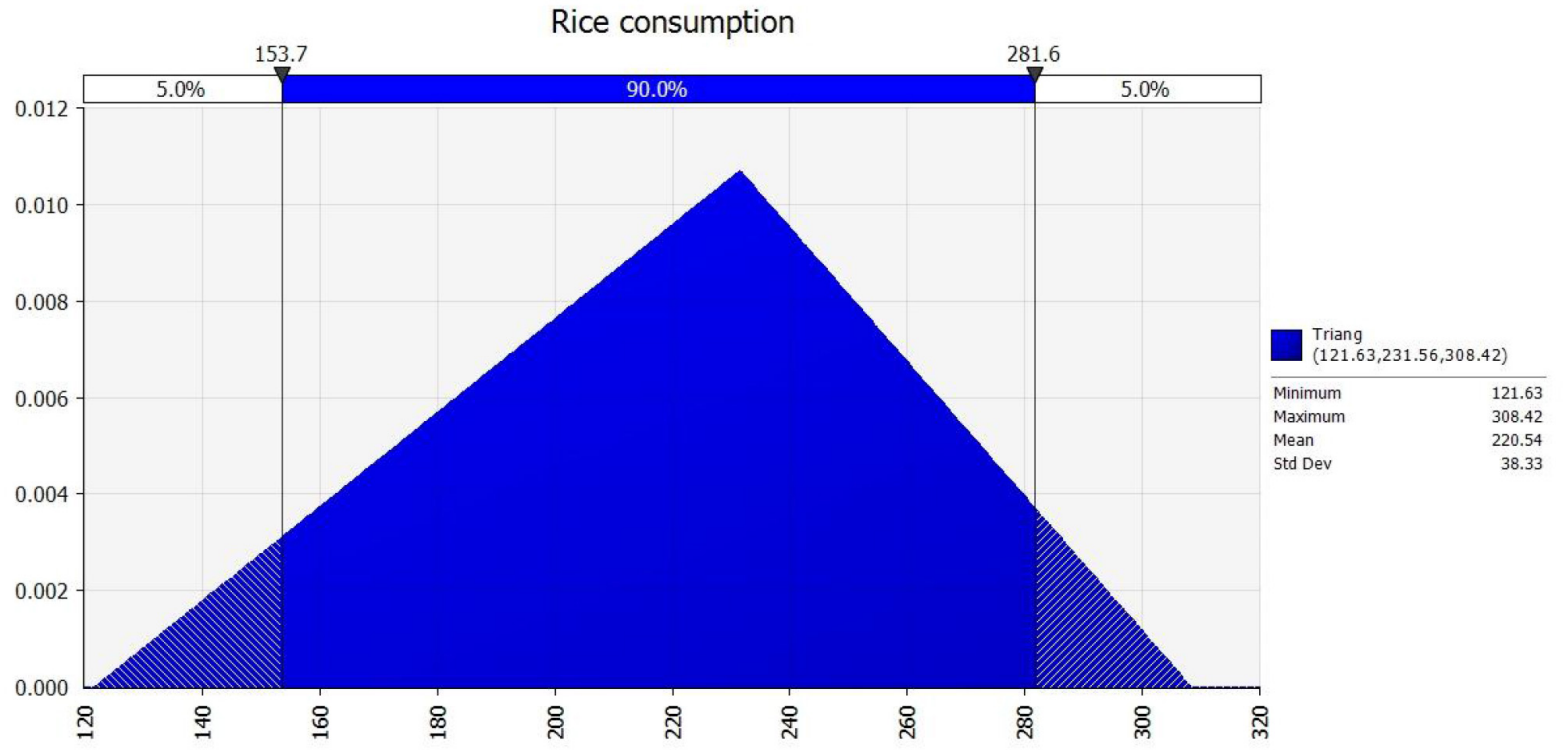
Fitted probability distribution of the distribution of residential consumption of rice and rice products.

Using Risk 7.6 software, cadmium content in 1,844 locally produced rice samples (2014–2020) was fitted using Chi-Squared, Anderson-Darling, and Kolmogorov-Smirnov tests (Figures 4, 5). A lognormal distribution [RiskLognorm (0.16781, 0.27126, RiskShift(-0.0015274))] best described Cd content, while rice consumption was modeled as a triangular distribution Triangular (121.63, 231.56, 308.42).

**Figure 6:**
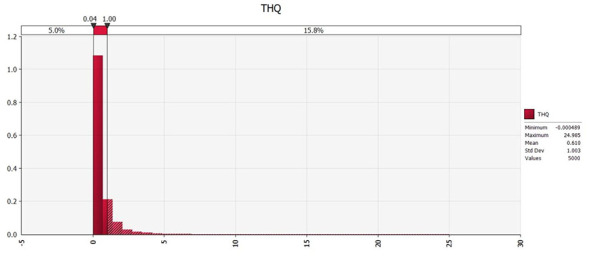
Probability distribution of THQ values for dietary risk hazard of cadmium in rice and rice products.

**Figure 7:**
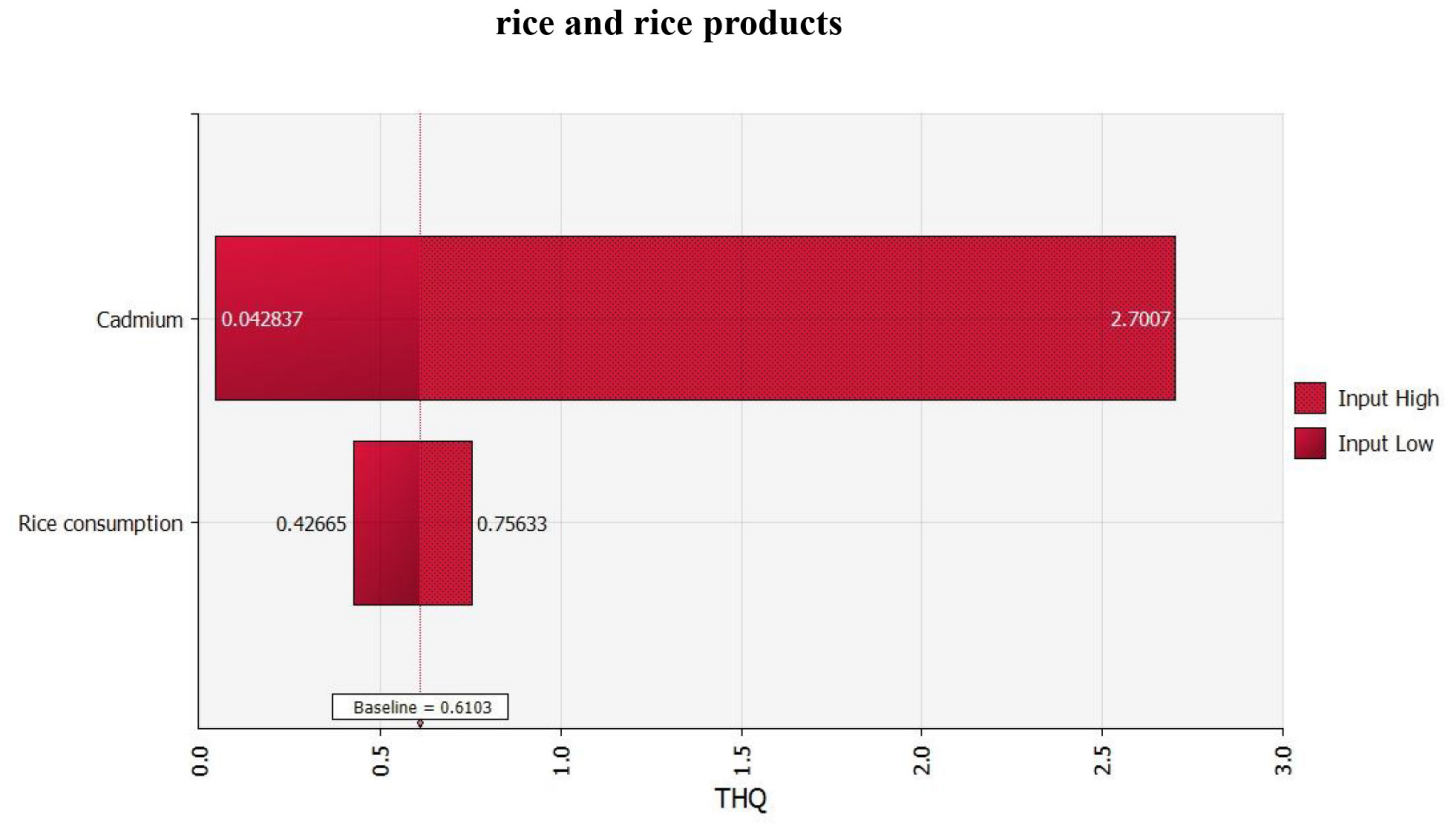
Sensitivity analysis of factors influencing the probability of the THQ value of dietary risk hazard for cadmium in rice and rice products (Inputs ranked by effect on output mean).

Based on the above distribution, the THQ results for cadmium in rice, calculated by simulating 5,000 iterations, are as follows (Figures 6, 7):

The Monte Carlo simulation revealed that the mean THQ for cadmium was 0.610, with 84.2% of the simulated population having THQ values below 1, indicating relatively low non-carcinogenic risk. However, 15.8% of the population had THQ ≥1, suggesting that a subset of the population may experience elevated potential exposure concerns from cadmium intake through rice consumption, rather than population-wide risk. From cadmium exposure through rice consumption. This highlights the need for continued cadmium monitoring and control in locally produced rice.

Sensitivity analysis identified cadmium concentration in rice as the most influential factor for health risk, emphasizing that reducing cadmium levels in rice would be the most effective strategy for lowering exposure-related risk.

## 4 Discussion

In the heavy metal and metalloids testing of rice in Nanning City, the highest level of cadmium contamination was identified, with a maximum detection value of 1.28 mg/kg, which is 6.4 times higher than the permissible limit set forth in China's health standards. This value is notably higher than the pooled mean cadmium concentration of 0.16 mg/kg reported across China in a recent systematic review (7), indicating that Nanning faces more severe contamination than many other rice-producing regions. The contamination of heavy metals and metalloids in rice was more prevalent in several locations, including Mashan, Wuming, Shanglin, and Binyang County. Concurrently, a survey of rice dietary consumption by residents in the area was conducted to calculate the non-carcinogenic and carcinogenic risks. The results indicated potential health concerns in specific subpopulations, particularly under higher exposure conditions. Guangxi is rich in mineral resources, particularly non-ferrous metals. Historical lead and zinc mining in Guangxi have contributed to persistent environmental contamination, particularly the elevated levels of cadmium (Cd) in rice-growing areas, likely reflecting a geogenic association between trace metals and metalloids (15). Some researchers have shown that cadmium is present in lead-zinc ores, and the Daxin lead-zinc mine, while recovering two types of metals, disposes of the remaining symbiotic metals in tailings. This practice has led to elevated cadmium concentrations in the tailings (16). Additionally, much of Guangxi's landscape is characterized by karst topography. Researchers Ji Wenbing and Thiago Nogueira Lucon have demonstrated that karst landscapes tend to exhibit higher levels of heavy metal contamination, including cadmium (17, 18). Previous studies have reported that cadmium contamination in agricultural soils is widespread across southern China, with regions characterized by karst geology and proximity to mining activities showing particularly elevated levels (19). The combined influence of naturally elevated soil cadmium baseline values and historical mining activities may contribute to the observed exceedance rates in Nanning City; however, as no direct soil or environmental measurements were conducted in the present study, this interpretation remains hypothetical and warrants further investigation.

In this study, cadmium contamination was substantial in certain areas. The deterministic assessment showed that average THQ values were below 1 in most regions, suggesting that typical exposure levels may not pose an appreciable non-carcinogenic risk. However, in specific subgroups—particularly children aged 6–17 years in Wuming County—THQ values exceeded 1, indicating potential health concern under higher exposure conditions. Under high exposure (P75), certain age groups, particularly children, exceeded the PTMI, indicating a non-negligible risk in these subpopulations.

Compared with the deterministic point estimation, the Monte Carlo probabilistic assessment provides a more comprehensive understanding of population-level risk by accounting for variability and uncertainty in both cadmium concentrations an and rice consumption rates (20). The deterministic assessment indicated that average THQ values were below the threshold of 1 in most regions of Nanning, whereas specific locations, especially in the vicinity of historical mining areas, exhibited THQ values above 1, which might suggest that overall population-level risk is limited; however, the probabilistic assessment revealed that 15.8% of the simulated population had THQ ≥1, a proportion that would not have been identified through point estimation alone. This discrepancy highlights the limitations of deterministic approaches, which tend to rely on single representative values and may underestimate risk in subpopulations with higher exposure. Furthermore, the sensitivity analysis confirmed that cadmium concentration in rice was a far more influential factor than rice consumption rate in determining health risk, indicating that reducing cadmium levels in rice should be prioritized as the primary mitigation strategy.

Since this study only considered rice and rice products as a single exposure pathway for cadmium, it did not account for the potential co-exposure to heavy metals and metalloids from other foods in the diet. This represents a limitation in assessing the overall risk of heavy metal exposure to the population. Furthermore, the study did not explicitly incorporate age-specific susceptibility differences in heavy metal exposure and toxicity. Zhan Liu (20) demonstrated that the probability of cancer risk is higher in children compared to adults due to their greater vulnerability. Future research should focus on collecting comprehensive age-stratified dietary consumption data to better characterize cumulative dietary exposure. This is necessary to refine the estimation of total health risk, as the current rice contamination dataset derives from annual routine monitoring programs rather than longitudinal dietary surveys, which limits the possibility of conducting long-term time-series exposure assessment.

Chronic cadmium exposure primarily targets the kidney, where cadmium accumulates in the proximal tubular cells and induces oxidative stress, leading to progressive decline in kidney function and eventually chronic kidney disease (21). Studies have demonstrated that even low-level cadmium exposure is associated with an increased risk of chronic kidney disease and albuminuria, suggesting that adverse effects may occur even at relatively low exposure levels. Beyond renal damage, chronic cadmium accumulation has also been linked to bone disease (2) including osteoporosis and osteomalacia, as illustrated by itai-itai disease historically documented in Japan, where cadmium-contaminated rice was the primary exposure source (3, 22). Population-based studies have further demonstrated that even low-level environmental cadmium exposure is associated with increased risk of bone demineralization and fractures (23). These findings underscore the importance of long-term health monitoring in populations with elevated dietary cadmium exposure, particularly in regions such as Nanning City where cadmium levels in locally produced rice exceeded national permissible limits.

The long-term accumulation of heavy metals and metalloids in the body can have lasting health consequences across the entire life cycle, and vulnerable populations such as pregnant women and newborns warrant particular attention due to the potential for cadmium to cross the placental barrier and affect fetal development (24). Standardized and sensitive detection methods should be implemented to better quantify population exposure over time. Based on the findings of this study, several targeted measures are recommended for Nanning City. Given the strong correlation between soil cadmium concentrations and rice cadmium accumulation documented across China (19), soil remediation efforts may be considered a priority in high-risk counties such as Wuming, Mashan, and Shanglin, where cadmium exceedance rates were the highest (25); second, the promotion of low-cadmium rice varieties and the adoption of clean cultivation practices should be encouraged in these regions; third, cadmium monitoring at market and supermarket levels should be strengthened to ensure that non-locally produced rice sold in the city also meets national safety standards; and finally, dietary guidance for children aged 6–17 years in high-risk areas should be provided, given that this group was identified as particularly vulnerable in the point assessment. Even though environmental heavy metal levels may gradually decline with ongoing pollution control efforts, the persistent presence of cadmium in locally grown rice continues to warrant monitoring and preventive intervention.

## 5 Strengths and limitations

Strengths: This study has several notable advantages. It is based on a large multi-year dataset (*n* >1,800) collected between 2014 and 2020, integrating both deterministic and probabilistic risk assessment approaches to evaluate population-level health risks. The use of GIS allowed for detailed spatial characterization of cadmium contamination in rice across Nanning City. Sensitivity analysis identified key exposure drivers, providing valuable guidance for targeted mitigation strategies. Given the high consumption of locally grown rice and the public health relevance of cadmium exposure in this region, the study offers important insights for risk management and policy planning.

Limitations: Several limitations should be acknowledged. Only rice and rice products were considered, excluding potential co-exposure from other dietary sources. Age-specific susceptibility, particularly for children, was not fully incorporated. Temporal trends could not be assessed despite the multi-year sampling period (2014–2020). Environmental measurements, including soil and water, were not conducted, limiting causal attribution of cadmium levels to mining activities and karst topography. Dietary intake data were not stratified by age, and Monte Carlo modeling lacked detailed reporting on distribution fitting. The use of the cadmium slope factor for carcinogenic risk assessment remains debated. Additionally, exposure calculations relied on cooked rice intake without continuous application of standard conversion factors, potentially introducing bias. Including these points enhances transparency and strengthens the scientific rigor of the study.

## Data Availability

The raw data supporting the conclusions of this article will be made available by the authors, without undue reservation.
